# The Impact of the COVID-19 Pandemic in Greece, 2020–January 2023: An Analysis Based on National Digital COVID-19 and Vaccination Registries

**DOI:** 10.3390/epidemiologia7050129

**Published:** 2026-09-17

**Authors:** Ivo Rakovac, Christos Triantafyllou, Vion Psiakis, Adela Galinovic, Ana Marques Domingues, Pavlos Myrianthefs, Joao Breda

**Affiliations:** 1 WHO Regional Office for Europe, DK-2100 Copenhagen, Denmark; rakovaci@who.int; 2 WHO Athens Quality of Care and Patient Safety Office, Ploutarchou 3, 10675 Athens, Greece; psiakisv@who.int (V.P.); adela.galinovic@outlook.com (A.G.); jesusa@who.int (A.M.D.); rodriguesdasilvabred@who.int (J.B.); 3 Department of Nursing, National and Kapodistrian University of Athens (NKUA), Papadiamantopoulou 123, 11527 Athens, Greece; pmiriant@nurs.uoa.gr

**Keywords:** COVID-19, mortality, risk factors, Greece, vaccination

## Abstract

Background/Objectives: From early 2020, Coronavirus Disease 2019 (COVID-19) pandemic spread rapidly worldwide, bringing unprecedented challenges. It severely disrupted health, social, and financial systems while exacerbating poverty and socioeconomic problems globally. This study aimed to investigate the characteristics and general health status—including age, comorbidities, and COVID-19 vaccination status—of SARS-CoV-2-infected patients as documented in Greek national registries, with a particular emphasis on COVID-19-related mortality. Methods: Anonymized individual-level data were obtained from the national digital registries of COVID-19 patients and vaccinations. Data extraction was initiated on 7 January 2023, and the linked anonymized dataset was provided to the research team on 18 January 2023. The data sets were linked by the e-Government Center for Social Security Services using the Greek social security number (AMKA). Data on comorbidities were estimated by, and extracted from, medicine prescriptions in outpatient settings using the standardized therapeutic prescribing protocol as defined by the Ministry of Health of the Hellenic Republic. Multivariable logistic regression was used to examine factors associated with COVID-19 death. Results: Of 33,740 people who died of COVID-19 infection, 70.0% (23,606) were not vaccinated. Sixty-seven point two percent (18,474) of individuals dying from COVID-19 were 70 years or older and not vaccinated. Hypertension was the most common comorbidity among patients who died from COVID-19 (67.1%). Among the 27,489 recorded COVID-19 deaths in individuals aged 70 years or older, unvaccinated individuals with two comorbidities accounted for 17.6% of deaths, while unvaccinated individuals with three comorbidities accounted for 16.7%. The multivariable analysis showed a much lower risk for people who had received two doses of the COVID-19 vaccine compared to those who are unvaccinated [odds ratio (OR) = 0.35; 95% CI: 0.33–0.36]. The existence of one or more comorbidities increased the risk of death (OR = 1.10; 95% CI: 1.09–1.11). Conclusions: Most COVID-19 fatalities in Greece occurred among unvaccinated individuals over 70 years of age. Mortality among vaccinated individuals was relatively low and was predominantly observed in those with multiple underlying health conditions. Thus, increasing Greece’s vaccination rate and addressing non-communicable diseases could significantly lower future COVID-19 deaths.

## 1. Introduction

Coronavirus Disease 2019 (COVID-19) is an infectious disease caused by the severe acute respiratory syndrome coronavirus 2 (SARS-CoV-2). The first cases were detected in December 2019 in China, after which the virus spread rapidly to many countries across the world. This led the World Health Organization (WHO) to declare a Public Health Emergency of International Concern on 30 January 2020 and to characterize the outbreak as a pandemic on 11 March 2020 [1,2,3]. By 10 June 2023, 676,609,955 cases and 6,881,955 deaths had been confirmed worldwide [4]. WHO estimates indicate that in 2020 and 2021 there were 14.8 million excess deaths, more than three times the number of COVID-19 deaths reported during this period [5].

Greece reported its first confirmed COVID-19 case on 26 February 2020 [6]. By 8 June 2023, a total of 22,124,235 COVID-19 vaccine doses had been administered in Greece. Of these, 7,935,564 people, representing 74.7% of the population, had received at least one dose, and 5,645,763 people, or 71.1% of those who had received at least one dose, had received two doses [7]. Additionally, a total of 7,122,161 booster shots had been given by the same date [8]. Vaccination uptake of two doses in the population aged 60 years and older plateaued around week 33 in the year 2021 with an uptake of 78.5%. Uptake then rose slowly, reaching 92.0% in this age group by early 2022. As of week 22 in the year 2023, the uptake of the first additional dose among people aged 60 years and older was 84.6%; for the second additional dose, 35.6% [7].

For the purpose of strategizing targeted interventions against the SARS-CoV-2 virus, it is important to understand the demographic and health status characteristics of those infected [9,10]. National registries provide a rich, comprehensive source of information for analysis, allowing insights into the composition of the population, the prevalence of comorbidities, as well as the vaccination status of the infected population against COVID-19 [11]. Many of the most relevant comorbidities in COVID-19 outcomes are non-communicable diseases, which represent a substantial and growing public health burden globally. Therefore, an investigation of this nature is key to unraveling the heterogeneous patterns of virus impact on various subsets of the population, as well as refining epidemiological models and forecasts [12].

Mortality associated with COVID-19 is of particular interest as it provides insights into the severity of the disease and the way in which the healthcare system is responding to it [13]. Information regarding mortality trends based on age, comorbidities and vaccination status is crucial in making healthcare policy decisions [10]. In other words, if higher mortality rates are observed among older individuals or those with certain comorbidities, this may assist in prioritizing preventive measures and healthcare resources for these vulnerable groups. In addition, examining the impact of vaccination on mortality rates will provide robust evidence regarding the effectiveness of vaccines, reinforcing public trust and encouraging vaccination uptake [14].

In Greece, there has yet to be an analysis of data from the national digital registry of COVID-19 patients focusing specifically on mortality. Therefore, the aim of this study was to investigate the characteristics and general health status—including age, comorbidities, and COVID-19 vaccination status—of SARS-CoV-2-infected patients as documented in national registries, with a particular emphasis on COVID-19-related mortality.

## 2. Materials and Methods

### 2.1. Data Sources, Methodology, and Definitions

Anonymized data were obtained from the national digital registries of COVID-19 patients and vaccinations after they had been linked by the e-Government Center for Social Security Services (IDIKA SA) using the Greek social security number (AMKA). Data on comorbidities were estimated by, and extracted from, medicine prescriptions in outpatient settings using the standardized therapeutic prescribing protocol as defined by the Ministry of Health of the Hellenic Republic (Table S1) [15].

Data extraction began on 7 January 2023, and the anonymized data were provided to the WHO Athens Quality of Care and Patient Safety Office and the WHO Country Office in Greece by IDIKA SA on 18 January 2023, following a request from the Ministry of Health of the Hellenic Republic.

COVID-19 wave periods were classified according to the date at which the predominant SARS-CoV-2 variant accounted for more than 50% of infections in Greece [16]. Accordingly, infections occurring before 28 June 2021 were assigned to the original/Alpha period, those occurring from 28 June to 19 December 2021 to the Delta period, and those occurring from 20 December 2021 onward to the Omicron period [16].

For the descriptive analyses, individuals with either no recorded COVID-19 vaccine doses or only one recorded dose were classified in the ‘not vaccinated’ category. For the multivariable logistic regression, individuals with exactly one recorded vaccine dose were excluded because of their small number; therefore, the reference category for vaccination status in the regression model comprised individuals with no recorded vaccine doses.

Individual-level dates of vaccination, SARS-CoV-2 infection, and death, as well as information on vaccine product/type, were not included in the anonymized analytic dataset available to the research team. Vaccination was therefore analyzed according to the recorded number of doses. Individual follow-up times, time since vaccination, and vaccination as a time-varying exposure could not be incorporated into the analysis.

#### National Digital Registry of COVID-19 Patients

The national digital registry of COVID-19 patients collates data of all individuals testing positive for COVID-19 and facilitates epidemiological data collection [17]. The registry aims to provide accurate and timely data to facilitate monitoring of the health situation and trends, evidence-based decision-making, improved coordination of healthcare services, and generally to inform measures for the protection of the population. In addition, the tool offers a telemedicine platform for patients infected with COVID-19 who are isolated at home.

The registry collects the following data and indicators: (1) COVID-19 incidence and admissions to hospitals; (2) number of positive polymerase chain reaction (PCR)/rapid tests; (3) epidemiological and medical records of patients; and (4) disease and recovery monitoring indicators from admission to discharge using COVID-19 patient files following WHO guidelines.

The national digital registry is a web-based platform that is linked to the nationwide citizen/refugee database and the electronic prescription system. It enables the collection, collation, and reporting of key data related to COVID-19, such as the total and daily numbers of COVID-19 hospitalized patients, number of COVID-19 deaths, number of COVID-19 ICU admissions, number of COVID-19 intubated patients, and respective outcomes. Additionally, it has helped to formulate therapy protocols in hospitals; to monitor the status of healthcare facilities; to allocate resources; to improve coordination and targeting of health services and interventions; to record epidemiological data; to plan for future needs; to improve coordination among several government agencies; and to enable health professionals to track patients’ progress through the telemedicine service and doctors to prescribe necessary medication [17].

### 2.2. Statistical Analysis

Summary statistics, such as frequency distributions and cross-tabulations, as well as medians, interquartile ranges, means, and standard deviations, were used to describe data. Categorical variables were tested using Pearson’s chi-squared test and continuous variables using the Kruskal–Wallis test (Tables S2–S4). Multivariable logistic regression was fitted to examine associations between the demographic and clinical characteristics available in the dataset, vaccination status, and the odds of death, while adjusting for the concurrent presence of multiple factors. The resulting odds ratios were interpreted as measures of association and not as causal estimates of vaccine effectiveness. For the multivariable regression analysis, comorbidity status was entered as a binary variable indicating the presence of one or more identified comorbidities versus none. The number of comorbidities was additionally examined descriptively in age-specific analyses. A nominal significance level of *p* value = 0.05 was set for all statistical analyses. Data analysis was performed using SPSS version 28 and R version 4.2.2 software with the Hmisc and RMS packages.

## 3. Results

### 3.1. COVID-19 Cases

The national digital registry contained 5,472,147 recorded SARS-CoV-2 infection episodes among 4,887,589 unique individuals (Table S2); 11.9% of individuals had more than one recorded infection. The median age of infected individuals was 43 years, and 47.8% of them were male. Most COVID-19 cases occurred in the 0–19 and 20–34 age groups (both 19.2%), followed by the 55–69 age group (17.5%). Four-fifths (80.0%) of infections occurred in 2022, 15.8% in 2021, and 1.6% in 2020.

Infections with different variants of SARS-CoV-2 had different transmissibility, severity, and immune escape; 5.2% of infections were caused by the original (Alpha) strain, 8.8% by the Delta strain, and 86.0% by the Omicron strain. Dyslipidaemia and hypertension were the most common comorbidities, with a prevalence of 24.2% and 18.6% respectively.

### 3.2. COVID-19 Deaths

A total of 33,740 deaths due to COVID-19 were registered in the national digital registry (Table S3), 54.7% of which occurred in males. The overall mortality rate was 0.6 per 100 cases. Four-fifths (81.5%) of individuals dying from COVID-19 were aged 70 years or older; 14.8% were 55–69 years; 2.8% were 45–54 years; 0.7% were 35–44 years; and 0.2% were 20–34 years. More than one-third (37.2%) of deaths were recorded in 2022, 46.5% in 2021, and 15.7% in 2020.

### 3.3. COVID-19 Mortality and Vaccination Against COVID-19

COVID-19 mortality, disaggregated by age group and number of doses of vaccines against COVID-19, is shown in Table 1. Of 33,740 people who died of COVID-19 infection, 70.0% were not vaccinated, 14.0% were vaccinated with two doses and 16.0% with three or more doses. More than two-thirds (67.2%) of individuals dying from COVID-19 were 70 years or older and not vaccinated.

**Figure 1:**
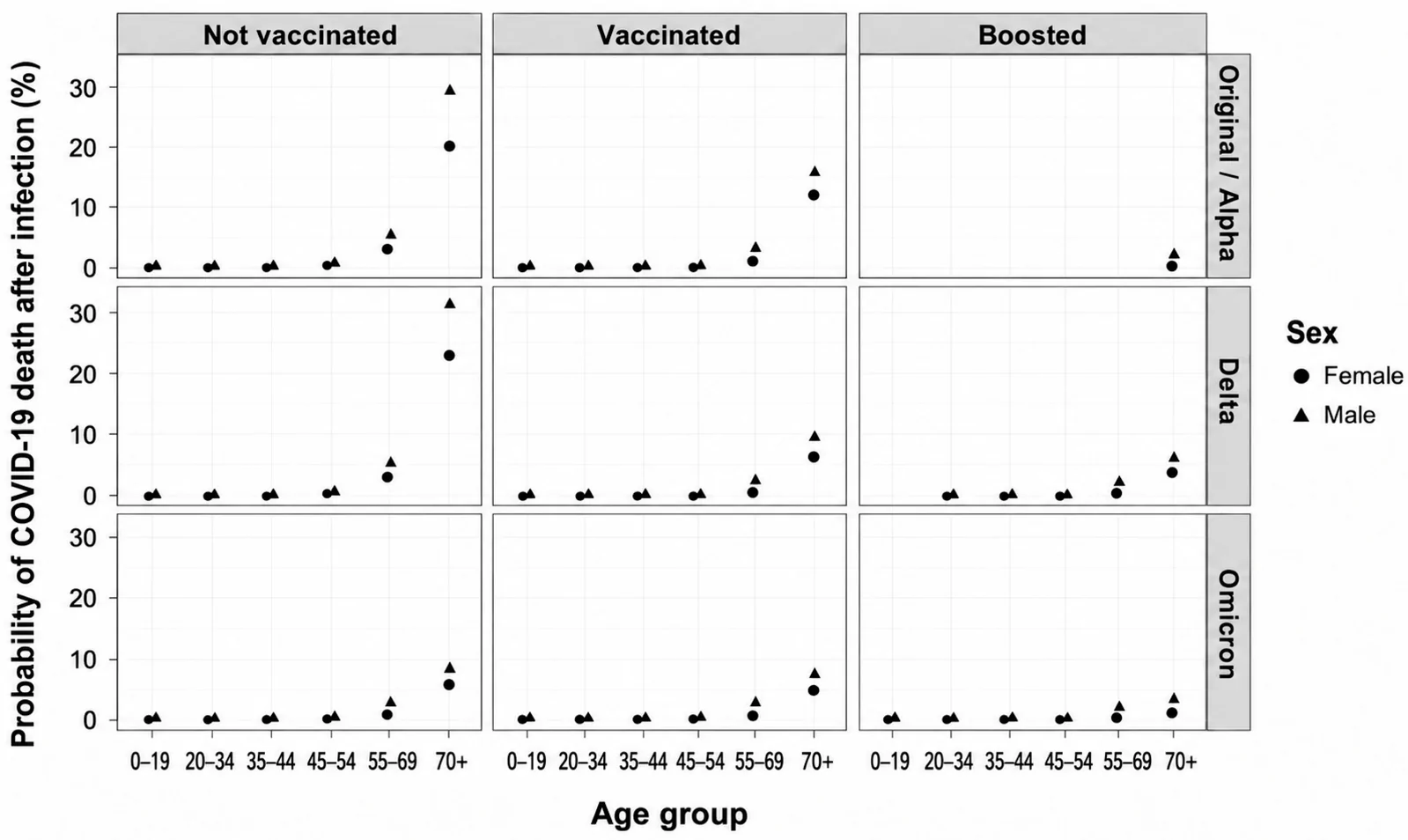
Probability of COVID-19 death following recorded SARS-CoV-2 infection in Greece, 2020–January 2023, according to age group, sex, vaccination category, and SARS-CoV-2 wave. ‘Not vaccinated’ refers to individuals with zero or one recorded vaccine dose in the descriptive analysis; ‘Vaccinated’ refers to individuals with two recorded doses; and ‘Boosted’ refers to individuals with three or more recorded doses.

The mortality rate from COVID-19 was 1.2 per 100 cases among those who had not been vaccinated, 0.4 per 100 among recipients of two doses, and 0.3 per 100 among recipients of three or more doses (Table S4). Mortality increased with age and the number of comorbidities and was considerably higher in the original/Alpha and Delta waves than in the Omicron wave (Figure 1 and Table S2). A large majority (70.0%) of individuals who died from COVID-19 were not vaccinated. Compared to those who recovered, decedents were significantly older (median age being 83 years) and had a considerable number of comorbidities (median 2) (Tables S2 and S3).

### 3.4. COVID-19 Mortality and Comorbidities

The median number of comorbidities among individuals dying from COVID-19 was 2.0 comorbidities per person (Table S2). One in eight (13.1%) of those dying from COVID-19 had no comorbidities identified through the prescription-based ascertainment method. The number of COVID-19 deaths, broken down by the most common comorbidities and age, is shown in Table 2 and Table S3. Overall, hypertension was the most common comorbidity among patients who died from COVID-19 (67.1%), followed by dyslipidaemia (62.7%), type 2 diabetes (35.1%) and coronary artery disease (22.8%) (Table 2 and Table S3). Among those aged 70 years and over who died from COVID-19, hypertension was the most common comorbidity (72.7%), followed by dyslipidaemia (66.4%), type 2 diabetes (37.6%) and coronary artery disease (25.3%) (Table 2 and Table S3).

### 3.5. Distribution of COVID-19 Deaths by Number of Vaccine Doses and Comorbidities: Age Group 70+

The distribution of COVID-19 deaths in the 70+ age group according to the number of comorbidities and number of vaccine doses is presented in Table 3. Over half of all individuals in the 70+ age group dying from COVID-19 had three (25.4%) or two comorbidities (25.3%). Within each of these groups, most individuals were not vaccinated: individuals aged 70+ who were not vaccinated and had two comorbidities represented 17.6% of all deaths due to COVID-19 in the age group, while such individuals with three comorbidities represented 16.7% of deaths. Most people in the 70+ age group who died from COVID-19 and had received two doses of the COVID-19 vaccine had either two comorbidities (3.5% of all deaths in the age group) or three (4.0% of deaths). Among those who had received three or more doses, they had either two comorbidities (4.1% of all deaths in the age group) or three (4.8% of deaths).

### 3.6. Distribution of COVID-19 Deaths by Number of Vaccine Doses and Comorbidities: Age Group 55–69

The distribution of COVID-19 deaths in the 55–69 age group according to the number of comorbidities and number of vaccine doses is presented in Table 4. Over half of all individuals in the 55–69 age group dying from COVID-19 had one comorbidity (23.2%) or none (27.1%). Within each of these groups, most individuals were not vaccinated (one dose or none). Individuals aged 55–69 who were not vaccinated and had no comorbidities represented 22.9% of all deaths due to COVID-19 in the age group, while such individuals with one comorbidity represented 19.1% of deaths. Among individuals who had received two vaccine doses, those with two comorbidities accounted for 2.6% of all deaths in this age group. Among individuals who had received three or more doses, those with one or two comorbidities accounted for 2.3% and 2.0% of all deaths in the age group, respectively.

### 3.7. Logistic Regression Analysis of the Risk of Dying from COVID-19

The results of a multivariable logistic regression analysis of the probability of death due to COVID-19, adjusted for age and a range of variables, are shown in Table 5. The analysis showed a higher mortality risk from COVID-19 for males, with an odds ratio (OR) of 1.72 (95% CI: 1.68–1.77), and a much lower risk for people who had received two doses of the COVID-19 vaccine compared to those who were unvaccinated, with an OR of 0.35 (95% CI: 0.33–0.36). The first additional (booster) dose further reduced the OR to 0.16 (95% CI: 0.15–0.17) compared to not vaccinated individuals, with the second booster reducing the OR even further to 0.09 (95% CI: 0.09–0.10). Previous infection also demonstrated a protective effect (OR = 0.42; 95% CI: 0.38–0.46). The existence of one or more comorbidities increased the risk of death, with an OR of 1.10 (95% CI: 1.09–1.11). The Omicron wave was considerably less lethal than the Delta wave (OR = 0.28; 95% CI: 0.27–0.29), while the lethality of the original/Alpha and Delta waves was similar (OR = 0.97; 95% CI: 0.94–1.01) and not statistically significant.

## 4. Discussion

This study presents, for the first time, an in-depth analysis of COVID-19 infection and vaccination registries in Greece, using linked, individual-level data.

The key finding of this study is that a large majority (70.0%) of individuals dying from COVID-19 in Greece in the period 2020–2023 were not vaccinated. The prevalence of comorbidities was very high among individuals dying from COVID-19, with a median of 2.0 comorbidities per person. The median age of deceased individuals was 83 years, and 81.5% of them were aged 70 years or older. More than two-thirds (67.2%) of individuals dying from COVID-19 were aged 70 years or older and not vaccinated. More than half of deaths (54.7%) occurred in males.

The odds of COVID-19 death increased markedly with age and were higher among males than females. Comorbidities, predominantly noncommunicable diseases, were common among individuals who died from COVID-19. In the multivariable analysis, the presence of at least one identified comorbidity was associated with higher odds of death. This finding is consistent with previously published evidence demonstrating the importance of underlying chronic conditions in adverse COVID-19 outcomes [18,19,20,21].

These findings have two important implications. First, as the Greek population is older and less healthy than the EU average [22,23,24,25], a higher-than-average mortality rate from COVID-19 could be expected, assuming other things are equal. Second, the importance of healthy aging, as well as prevention and high-quality care for NCDs and other conditions, is highlighted in order to increase resilience and reduce the adverse impacts of future pandemics. Therefore, it is important to adopt a dual-track approach: tackling COVID-19 and other health issues, most notably NCDs, simultaneously, while increasing investment in public health to make populations healthier across the life course. Prevention and control of NCDs are also highly cost-effective and give an excellent return on investment [26,27,28].

Vaccines were remarkably effective, significantly reducing the risk of death from COVID-19 across all waves and age groups and for both sexes in Greece. The effect was further increased by first and second booster vaccinations. The effectiveness and safety of COVID-19 vaccines have been widely demonstrated [14,29,30]. As vaccine effectiveness wanes over time, especially among elderly people and people with comorbidities, booster vaccine doses are key tools in protecting the most vulnerable [31,32]. WHO’s Strategic Advisory Group of Experts on Immunization recently issued updated guidance on COVID-19 vaccination, emphasizing the role of boosters for the most vulnerable groups [33]. All the above evidence and results illustrate the crucial role of vaccination in preventing severe disease and death from COVID-19 and highlight vaccination as a critical measure for pandemic control. Therefore, high vaccination uptake, including boosters, is absolutely crucial to reducing COVID-19 mortality, particularly in vulnerable populations. Interventions to increase vaccination uptake, including booster uptake, should continue to be considered a priority.

A case fatality ratio of 0.6 deaths per 100 infections in Greece is relatively low in the European context [34,35] and could be indicative of a strong surveillance system and adequate quality of care, especially given that the Greek population is older and less healthy than the European average. A relatively low reported positivity rate [36] and a close match between excess and reported COVID-19 mortality in the years 2020 and 2021 are also indicative of a strong surveillance system [5]. However, an in-depth assessment of the surveillance system and healthcare quality is needed to confirm this.

The strengths of this study include the large number of observations based on census data, as well as the rich and comprehensive dataset. Moreover, triangulation with other publicly available data sources confirmed that the data on infections and deaths in Greece were comparable to those reported through other channels without major discrepancies [7,35,37]. Greece adopted a definition of death from COVID-19 for surveillance purposes that was aligned with the WHO definition [38] and included all reported deaths from COVID-19 in the registry, irrespective of the time elapsed since infection or a positive test; this contrasts with the practice of some other European countries, which used a maximum interval of 28 days between the dates of a positive test and death [39].

A major limitation of the study is the approach used to approximate the presence of comorbidities based on medical prescriptions in outpatient settings, as this excludes undiagnosed cases and underestimates the prevalence of diagnosed conditions that are treated in hospitals only and are not treated with medicines. In addition, data linkage was performed using the Greek social security number (AMKA), meaning that completeness of the study population depends on the presence and accurate recording of AMKA across administrative systems. This may have resulted in incomplete linkage for certain individuals, potentially leading to underrepresentation of population groups with fragmented or incomplete administrative records, and introducing selection bias if such groups differ systematically in health status or mortality risk.

With the data available in the COVID-19 registry or from publicly available data sources, it is not possible to clarify what proportion of infected people died from COVID-19, as distinct from those who died with COVID-19, i.e., cases where COVID-19 was not the underlying cause of death. Evidence from Denmark [40] and the United Kingdom [41] suggests that up to the end of the Delta wave when the availability of vaccines was limited, almost all deaths reported through registries were indeed due to COVID-19 according to the WHO definition [42]. However, in the Omicron wave, which was characterized by higher transmissibility and lower lethality compared to Delta, as well as high uptake of vaccines, more than 50% of deaths included in registries were deaths with, but not due to, COVID-19, i.e., COVID-19 was not the underlying cause of death. It is also possible that some deaths due to COVID-19 were not included in the registry, as people were not tested after they had died. Moreover, while the linked registries provided a rich source of information on demographic characteristics, vaccination status, and comorbidities, additional variables not available in the registries may also contribute to differences in COVID-19 mortality outcomes.

A further major limitation concerns the absence of detailed temporal and vaccine-specific information in the anonymized analytic dataset. Individual-level dates of vaccination, SARS-CoV-2 infection, and death were not available to the research team. Consequently, vaccination could not be modelled as a time-varying exposure, and individual-level intervals between vaccination and infection, between infection and death, and time since vaccination could not be assessed. This also precluded the application of time-to-event methods and a formal assessment of seasonal patterns. Information on vaccine product/type was also unavailable, and vaccination was therefore analyzed according to the recorded number of doses only. Accordingly, the odds ratios derived from the multivariable logistic regression should be interpreted as measures of association rather than causal estimates of vaccine effectiveness.

The multivariable model treated comorbidity status as a binary variable; therefore, the regression estimate does not capture potential gradients associated with the number, type, or combinations of comorbidities.

A further limitation is that formal residual diagnostics and goodness-of-fit assessments were not available for the multivariable logistic regression model. Although the very large nationwide dataset provides substantial statistical precision, the absence of formal model-diagnostic measures limits assessment of model calibration and overall fit.

Lastly, it should be acknowledged that this was the first attempt to analyze linked data from the registries; hence additional and regular analyses are needed, with a focus on quality of care and improving data utility and quality. If relevant data were available, it would be interesting to analyze inequalities by socioeconomic status as well as by uptake of antiviral treatments.

## 5. Conclusions

In conclusion, the majority of people (67.2%) who died from COVID-19 in Greece were aged 70 years or older and not vaccinated against COVID-19. Moreover, the prevalence of comorbidities was high, with hypertension being the most common comorbidity among people who died from COVID-19 (67.1%), followed by dyslipidaemia, type 2 diabetes, and coronary artery disease. Furthermore, most of the people who died from COVID-19 belonged to the 70+ age group and had two or three comorbidities. Lastly, most people who died from COVID-19 and had received two or more doses of COVID-19 vaccines had either two or three comorbidities, in particular NCDs.

These findings suggest that vaccination uptake in Greece (including booster uptake) could be strengthened through targeted outreach strategies such as primary care–based vaccination, mobile vaccination units, and reminder systems for older adults. Simultaneously, Greece would greatly benefit from comprehensive public health initiatives addressing NCDs and risk factors (diet, physical activity, alcohol, and tobacco use). In particular, programs targeting hypertension and other cardiovascular conditions, diabetes, and obesity (e.g., population screening) should be strengthened, and the quality of care improved, in order to achieve a healthier and more resilient population for the future.

Moreover, it is suggested that consideration should be given to performing an after-action review (AAR) [43], to consider and assess further lines of inquiry beyond the analysis of data from COVID-19 registries and to share lessons learned. An AAR could focus on areas of particular interest, such as quality of care, surveillance, and public health and social measures. In addition, a twinning exercise could also be considered, which would involve structured collaboration in order to exchange best practices and improve data-driven public health decision-making.

## Figures and Tables

**Table 1:** Number of COVID-19 deaths in Greece, 2020–January 2023, by age and number of vaccine doses.

Age Group (Years)	0–19	20–34	35–44	45–54	55–69	70+	Total (n = 33,740)
Not vaccinated (n, %)	8 (100.0)	63 (87.5)	208 (86.3)	822 (87.4)	4031 (80.8)	18,474 (67.2)	23,606 (70.0)
Two doses (n, %)	0 (0.0)	9 (12.5)	21 (8.7)	69 (7.3)	491 (9.8)	4131 (15.0)	4721 (14.0)
Three or more doses (n, %)	0 (0.0)	0 (0.0)	12 (5.0)	50 (5.3)	467 (9.4)	4884 (17.8)	5413 (16.0)

**Table 2:** Number of COVID-19 deaths in Greece, 2020–2023, by age and comorbidity.

Age Group (Years)	0–19	20–34	35–44	45–54	55–69	70+	Total
Number of deaths per age group	8	72	241	941	4989	27,489	33,740
Hypertension (n, %)	0 (0)	9 (12.7)	34 (14.2)	243 (25.8)	2383 (47.8)	19,985 (72.7)	22,654 (67.1)
Dyslipidaemia (n, %)	0 (0)	6 (8.5)	30 (12.5)	256 (27.2)	2604 (52.2)	18,261 (66.4)	21,157 (62.7)
Diabetes mellitus—type 2 (n, %)	0 (0)	7 (9.7)	18 (7.5)	139 (14.8)	1360 (27.3)	10,332 (37.6)	11,856 (35.1)
Coronary artery disease (n, %)	0 (0)	0 (0)	4 (1.7)	29 (3.1)	701 (14.1)	6944 (25.3)	7678 (22.8)
Atrial fibrillation (n, %)	0 (0)	0 (0)	5 (2.1)	10 (1.1)	235 (4.7)	5391 (19.6)	5641 (16.7)
COPD (n, %)	1 (12.5)	0 (0)	10 (4.2)	30 (3.2)	442 (8.9)	3561 (13%)	4044 (12.0)
Cardiac failure (n, %)	0 (0)	0 (0)	6 (2.5)	11 (1.2)	186 (3.7)	2297 (8.4)	2500 (7.4)

Figures in brackets indicate the percentage of all deaths due to COVID-19 within each age group.

**Table 3:** Number of COVID-19 deaths among individuals aged 70+ years in Greece, 2020–January 2023, by number of comorbidities and number of vaccine doses.

Age Group 70+	Number of Vaccine Doses Against COVID-19			Total Number of Deaths
Number of Comorbidities	Not Vaccinated	Two Doses	Three or More Doses	
0 (n, %)	1821 (6.6)	280 (1.0)	277 (1.0)	2378 (8.7)
1 (n, %)	3125 (11.4)	615 (2.2)	625 (2.3)	4365 (15.9)
2 (n, %)	4842 (17.6)	974 (3.5)	1128 (4.1)	6944 (25.3)
3 (n, %)	4587 (16.7)	1099 (4.0)	1307 (4.8)	6993 (25.4)
4 (n, %)	2723 (9.9)	696 (2.5)	927 (3.4)	4346 (15.8)
5 (n, %)	1021 (3.7)	317 (1.2)	454 (1.7)	1792 (6.5)
6 (n, %)	289 (1.1)	120 (0.4)	127 (0.5)	536 (1.9)
7+ (n, %)	66 (0.2)	30 (0.1)	39 (0.1)	135 (0.5)
Number of deaths	18,474 (67.2)	4131 (15.0)	4884 (17.8)	27,489

Figures in brackets indicate the percentage of all deaths at age 70+ years due to COVID-19 (27,489).

**Table 4:** Number of COVID-19 deaths among individuals aged 55–69 years in Greece, 2020–January 2023, by number of comorbidities and number of vaccine doses.

Age Group 55–69 Years	Number of Vaccine Doses Against COVID-19			Total Number of Deaths
Number of Comorbidities	Not Vaccinated	Two Doses	Three or More Doses	
0 (n, %)	1144 (22.9)	113 (2.3)	92 (1.8)	1349 (27.1)
1 (n, %)	955 (19.1)	90 (1.8)	113 (2.3)	1158 (23.2)
2 (n, %)	921 (18.5)	130 (2.6)	98 (2.0)	1149 (23.0)
3 (n, %)	607 (12.2)	75 (1.5)	80 (1.6)	762 (15.3)
4 (n, %)	284 (5.7)	57 (1.1)	56 (1.1)	397 (7.9)
5 (n, %)	90 (1.8)	21 (0.4)	22 (0.4)	133 (2.7)
6 (n, %)	27 (0.5)	5 (0.1)	5 (0.1)	37 (0.7)
7+ (n, %)	3 (0.1)	0 (0.0)	1 (0.0)	4 (0.1)
Number of deaths	4031 (80.8)	491 (9.8)	467 (9.4)	4989

Percentages are calculated using all 4989 recorded COVID-19 deaths among individuals aged 55–69 years as the denominator; they represent the distribution of deaths and not mortality risks.

**Table 5:** Results of a multivariable logistic regression analysis of the risk of dying from COVID-19 in Greece, 2020–January 2023, adjusted for age and a range of variables.

Variable	OR	95% CI	*p* Value
Male vs. female	1.72	1.68–1.77	<0.001
Two COVID-19 vaccine doses vs. no recorded doses	0.35	0.33–0.36	<0.001
First additional vaccination vs. no recorded doses	0.16	0.15–0.17	<0.001
Second additional vaccination vs. no recorded doses	0.09	0.09–0.10	<0.001
Previous infection	0.42	0.38–0.46	<0.001
One comorbidity or more	1.10	1.09–1.11	<0.001
Original/Alpha vs. Delta wave	0.97	0.94–1.01	0.125
Omicron vs. Delta wave	0.28	0.27–0.29	<0.001

Note: COVID-19, coronavirus disease 2019; CI, confidence interval; OR, odds ratio.

## Data Availability

The datasets used and/or analysed during the current study are available from the corresponding author on reasonable request.

## Supplementary Materials

The following supporting information can be downloaded at: https://www.mdpi.com/article/10.3390/epidemiologia7050129/s1, Table S1: Diseases for which the standardized therapeutic prescribing protocol, as defined by the Ministry of Health, was used to identify comorbidities among patients with COVID-19; Table S2: Descriptive statistics of individuals registered in the national digital COVID-19 registry, stratified by reported outcome (recovery or death from COVID-19); Table S3: Descriptive statistics of individuals who died from COVID-19, stratified by age group; Table S4: Descriptive statistics of individuals registered in the national digital COVID-19 registry, stratified by vaccination status according to the number of vaccine doses received.

## Abbreviations

The following abbreviations are used in this manuscript:

AAR	After-action review
AMKA	Greek social security number
CI	Confidence interval
COPD	Chronic obstructive pulmonary disease
COVID-19	Coronavirus disease 2019
ECDC	European Centre for Disease Prevention and Control
EU	European Union
ICU	Intensive care unit
IDIKA S.A.	e-Government Center for Social Security Services S.A.
NCD	Noncommunicable disease
OR	Odds ratio
PCR	Polymerase chain reaction
SARS-CoV-2	Severe acute respiratory syndrome coronavirus 2
SPSS	Statistical Package for the Social Sciences
WHO	World Health Organization
