# Supplementary material for: The Impact of the COVID-19 Pandemic in Greece, 2020–January 2023: An Analysis Based on National Digital COVID-19 and Vaccination Registries
Source: Epidemiologia (Basel). 2026 Sep 17;7(5):129. doi: 10.3390/epidemiologia7050129 (PMC13600023; doi:10.3390/epidemiologia7050129)
Supplement: Supplementary file 1 [file epidemiologia-07-00129-s001.zip › epidemiologia-4512708-supplementary.pdf]

Supplementary material

Table S1. Diseases for which the standardized therapeutic prescribing protocol, as defined by the Ministry of Health, was used to extract data on COVID-19 patients' comorbidities**.**

1	Chronic obstructive pulmonary disease (COPD)
2	Ulcerative colitis
3	Peptic ulcer
4	Dyslipidaemia
5	Dyslipidaemia – PCSK9 inhibitors
6	Hypertension
7	Cardiac failure
8	Atrial fibrillation
9	Coronary artery disease
10	Relapsing remitting multiple sclerosis
11	Advanced Parkinson disease
12	Alzheimer disease
13	Axial spondylarthritis
14	Rheumatoid arthritis
15	Diabetes mellitus – neonatal
16	Gestational diabetes mellitus
17	Diabetes mellitus – type 1
18	Diabetes mellitus – type 2
19	Diabetes mellitus – insulin deficiency after medical procedures
20	Diabetes mellitus – other types
21	Thromboembolic disease – treatment in cancer patients

Table S2. Descriptive statistics of individuals registered in the COVID-19 national digital registry, stratified by reported status as recovered or died from COVID-19**.**

	Overall<br>*n* = 4,887,589	Recovered<br>*n* = 4,853,849	Died from COVID-19<br>*n* = 33,740	*p* value
**Age (years)**	25.0 43.0 58.0[a]<br>(42.4 ±21.6)[b]	25.0 42.0 58.0[a]<br>(42.2 ±21.4)[b]	73.0 83.0 89.0[a]<br>(80.0 ±12.1)[b]	< 0.001
**Age group: 0–19 years**	18.9% (923,699)	19.0% (923,691)	0.0% (8)	< 0.001
**20–34 years**	18.5% (903,164)	18.6% (903,092)	0.2% (72)	
**35–44 years**	15.9% (778,423)	16.0% (778,182)	0.7% (241)	
**45–54 years**	16.6% (810,125)	16.7% (809,184)	2.8% (941)	
**55–69 years**	18.1% (885,379)	18.1% (880,390)	14.8% (4989)	
**70+ years**	12.0% (586,799)	11.5% (559,310)	81.5% (27,489)	
**Sex: male**	47.8% (2,336,377)	47.8% (2,317,919)	54.7% (18,458)	< 0.001
**Previous infections: 0**	88.1% (4 303 777)	88.0% (4,270,502)	98.6% (33,275)	< 0.001
**1**	11.9% (583,439)	12.0% (582,977)	1.4% (462)	
**2+**	0.0% (373)	0.0% (370)	0.0% (3)	
**Number of comorbidities: 0**	67.9% (3,317,417)	68.3% (3,312,982)	13.1% (4435)	< 0.001
**1**	13.9% (679,141)	13.9% (673,324)	17.2% (5817)	
**2**	9.5% (463,981)	9.4% (455,723)	24.5% (8258)	
**3**	5.7% (276,966)	5.5% (269,136)	23.2% (7830)	
**4**	2.2% (109,029)	2.1% (104,267)	14.1% (4762)	
**5**	0.7% (31,883)	0.6% (29,957)	5.7% (1926)	
**6**	0.2% (7622)	0.1% (7049)	1.7% (573)	
**7**	0.0% (1550)	0.0% (1411)	0.4% (139)	
**Number of comorbidities**	0.0 0.0 1.0[a]<br>(0.6 ±1.1)[b]	0.0 0.0 1.0[a]<br>(0.6 ±1.1)[b]	1.0 2.0 3.0[a]<br>(2.3 ±1.5)[b]	< 0.001
**Year: 2020**	1.6% (78,854)	1.5% (73,544)	15.7% (5310)	< 0.001
**2021**	15.8% (772,359)	15.6% (756,686)	46.5% (15,673)	
**2022**	80.0% (3,912,162)	80.3% (3,899,603)	37.2% (12,559)	
**2023**	2.5% (124,213)	2.6% (124,015)	0.6% (198)	
**Wave: Original/Alpha**	5.2% (254,290)	5.0% (242,009)	36.4% (12,281)	< 0.001
**Delta**	8.8% (429,567)	8.7% (421,630)	23.5% (7937)	
**Omicron**	86.0% (4,203,731)	86.3% (4,190,209)	40.1% (13,522)	
**COPD**	2.2% (109,836)	2.2% (105,792)	12.0% (4044)	< 0.001
**Ulcerative colitis**	0.3% (14,352)	0.3% (14,234)	0.3% (118)	0.056
**Peptic ulcer**	0.2% (10,658)	0.2% (10,541)	0.3% (117)	< 0.001
**Dyslipidaemia**	24.2% (1,181,224)	23.9% (1,160,075)	62.7% (21,149)	< 0.001

**Dyslipidaemia – PCSK9 inhibitors**	0.0% (1664)	0.0% (1656)	0.0% (8)	0.302
**Hypertension**	18.6% (907,610)	18.2% (884,956)	67.1% (22,654)	< 0.001
**Cardiac failure**	0.7% (36,240)	0.7% (33,740)	7.4% (2500)	< 0.001
**Atrial fibrillation**	2.2% (108,067)	2.1% (102,426)	16.7% (5641)	< 0.001
**Coronary artery disease**	4.1% (200,202)	4.0% (192,524)	22.8% (7678)	< 0.001
**Relapsing remitting multiple sclerosis**	0.0% (1139)	0.0% (1133)	0.0% (6)	0.505
**Advanced Parkinson disease**	0.2% (8059)	0.1% (7177)	2.6% (882)	< 0.001
**Alzheimer disease**	0.1% (4944)	0.1% (4475)	1.4% (469)	< 0.001
**Axial spondylarthritis**	0.2% (10,916)	0.2% (10,880)	0.1% (36)	< 0.001
**Rheumatoid arthritis**	0.7% (32,769)	0.7% (32,102)	2.0% (667)	< 0.001
**Diabetes mellitus – neonatal**	0.0% (19)	0.0% (18)	0.0% (1)	0.016
**Gestational diabetes mellitus**	0.2% (11,745)	0.2% (11,743)	0.0% (2)	< 0.001
**Diabetes mellitus – type 1**	0.0% (502)	0.0% (474)	0.1% (28)	< 0.001
**Diabetes mellitus – type 2**	8.5% (413,057)	8.3% (401,201)	35.1% (11,856)	< 0.001
**Diabetes mellitus – insulin deficiency after medical procedures**	0.4% (21,484)	0.4% (20,810)	2.0% (674)	< 0.001
**Diabetes mellitus – other types**	0.3% (12,614)	0.3% (12,337)	0.8% (277)	< 0.001
**Thromboembolic disease – treatment in cancer patients**	0.0% (90)	0.0% (86)	0.0% (4)	< 0.001
**Treatment of venous thromboembolic disease**	0.1% (2852)	0.1% (2750)	0.3% (102)	< 0.001
**Thromboembolic disease – prevention in cancer patients**	0.0% (277)	0.0% (260)	0.1% (17)	< 0.001

[a] Quartiles: 25th, 50th, and 75th percentile; [b] mean ± standard deviation.

Table S3. Descriptive statistics of individuals reported as dying from COVID-19, stratified by age.

**Age group (years)**	**0–19** ***n* = 8**	**20–34** ***n* = 72**	**35–44** ***n* = 241**	**45–54** ***n* = 941**	**55–69** ***n* = 4989**	**70+** ***n* = 27,489**	**All** ***n* = 33,740**	***p* value**
**Age (years)**	3.0 9.5 16.5[a] (9.9 ± 7.4)[b]	27.0 30.0 33.0[a] (29.3 ± 3.8)[b]	38.0 41.0 43.0[a] (40.5 ± 2.7)[b]	48.0 51.0 53.0 (50.4 ± 2.8)[b]	60.0 64.0 67.0[a] (63.5 ± 4.1)[b]	78.0 85.0 90.0[a] (84.5 ± 7.5)[b]	73.0 83.0 89.0[a] (80.0 ±12.1)[b]	< 0.001
**Sex: male**	37.5% (3)	70.8% (51)	67.2% (162)	67.7% (637)	64.2% (3201)	52.4% (14,404)	54.7% (18,458)	< 0.001
**Vaccination: Not vaccinated**	100.0% (8)	87.5% (63)	86.3% (208)	87.4% (822)	80.8% (4031)	67.2% (18474)	70.0% (23,606)	< 0.001
**Two doses**	0.0% (0)	12.5% (9)	8.7% (21)	7.3% (69)	9.8% (491)	15.0% (4131)	14.0% (4721)	
**Three or more doses**	0.0% (0)	0.0% (0)	5.0% (12)	5.3% (50)	9.4% (467)	17.8% (4884)	16.0% (5413)	
**Number of comorbidities: 0**	87.5% (7)	79.2% (57)	68.0% (164)	51.0% (480)	27.0% (1349)	8.7% (2378)	13.1% (4435)	< 0.001
**1**	12.5% (1)	9.7% (7)	18.3% (44)	25.7% (242)	23.2% (1158)	15.9% (4365)	17.2% (5817)	
**2**	0.0% (0)	8.3% (6)	9.1% (22)	14.6% (137)	23.0% (1149)	25.3% (6944)	24.5% (8258)	
**3**	0.0% (0)	2.8% (2)	3.7% (9)	6.8% (64)	15.3% (762)	25.4% (6993)	23.2% (7830)	
**4**	0.0% (0)	0.0% (0)	0.4% (1)	1.9% (18)	8.0% (397)	15.8% (4346)	14.1% (4762)	
**5**	0.0% (0)	0.0% (0)	0.4% (1)	0.0% (0)	2.7% (133)	6.5% (1792)	5.7% (1926)	
**6**	0.0% (0)	0.0% (0)	0.0% (0)	0.0% (0)	0.7% (37)	1.9% (536)	1.7% (573)	
**7+**	0.0% (0)	0.0% (0)	0.0% (0)	0.0% (0)	0.1% (4)	0.5% (135)	0.4% (139)	
**Number of comorbidities**	0.0 0.0 0.0[a] (0.1 ±0.4)[b]	0.0 0.0 0.0[a] (0.3 ±0.8)[b]	0.0 0.0 1.0[a] (0.5 ±0.9)[b]	0.0 0.0 1.0[a] (0.8 ±1.0)[b]	0.0 1.0 3.0[a] (1.7 ±1.4)[b]	2.0 3.0 3.0[a] (2.5 ±1.5)[b]	1.0 2.0 3.0[a] (2.3 ±1.5)[b]	< 0.001
**Number of comorbidities: 0**	87.5% (7)	79.2% (57)	68.0% (164)	51.0% (480)	27.0% (1349)	8.7% (2378)	13.1% (4435)	< 0.001
**1–2**	12.5% (1)	18.1% (13)	27.4% (66)	40.3% (379)	46.2% (2307)	41.1% (11,309)	41.7% (14,075)	
**3+**	0.0% (0)	2.8% (2)	4.6% (11)	8.7% (82)	26.7% (1333)	50.2% (13 802)	45.1% (15,230)	
**Not vaccinated or at least one comorbidity**	100.0% (8)	91.7% (66)	90.9% (219)	94.4% (888)	95.9% (4784)	98.0% (26,932)	97.5% (32,897)	< 0.001
**Year: 2020**	0.0% (0)	12.5% (9)	17.0% (41)	14.0% (132)	16.6% (827)	15.6% (4301)	15.7% (5310)	< 0.001
**2021**	50.0% (4)	58.3% (42)	59.8% (144)	62.8% (591)	57.4% (2863)	43.8% (12,029)	46.5% (15,673)	
**2022**	50.0% (4)	29.2% (21)	23.2% (56)	22.8% (215)	25.8% (1286)	39.9% (10,977)	37.2% (12,559)	
**2023**	0.0% (0)	0.0% (0)	0.0% (0)	0.3% (3)	0.3% (13)	0.7% (182)	0.6% (198)	
**Wave: Original/Alpha**	37.5% (3)	25.0% (18)	36.5% (88)	36.3% (342)	41.0% (2047)	35.6% (9783)	36.4% (12 281)	< 0.001
**Delta**	12.5% (1)	40.3% (29)	37.8% (91)	37.4% (352)	29.8% (1487)	21.7% (5977)	23.5% (7937)	
**Omicron**	50.0% (4)	34.7% (25)	25.7% (62)	26.2% (247)	29.2% (1455)	42.7% (11,729)	40.1% (13,522)	
**COPD**	12.5% (1)	0.0% (0)	4.1% (10)	3.2% (30)	8.9% (442)	13.0% (3561)	12.0% (4044)	< 0.001

**Ulcerative colitis**	0.0% (0)	0.0% (0)	0.8% (2)	0.5% (5)	0.4% (18)	0.3% (93)	0.3% (118)	0.718
**Peptic ulcer**	0.0% (0)	0.0% (0)	0.4% (1)	0.5% (5)	0.5% (25)	0.3% (86)	0.3% (117)	0.348
**Dyslipidaemia**	0.0% (0)	8.3% (6)	12.4% (30)	27.2% (256)	52.1% (2600)	66.4% (18,257)	62.7% (21,149)	< 0.001
**Dyslipidaemia – PCSK9 inhibitors**	0.0% (0)	0.0% (0)	0.0% (0)	0.0% (0)	0.1% (4)	0.0% (4)	0.0% (8)	0.157
**Hypertension**	0.0% (0)	12.5% (9)	14.1% (34)	25.8% (243)	47.8% (2383)	72.7% (19,985)	67.1% (22,654)	< 0.001
**Cardiac failure**	0.0% (0)	0.0% (0)	2.5% (6)	1.2% (11)	3.7% (186)	8.4% (2297)	7.4% (2500)	< 0.001
**Atrial fibrillation**	0.0% (0)	0.0% (0)	2.1% (5)	1.1% (10)	4.7% (235)	19.6% (5391)	16.7% (5641)	< 0.001
**Coronary artery disease**	0.0% (0)	0.0% (0)	1.7% (4)	3.1% (29)	14.1% (701)	25.3% (6944)	22.8% (7678)	< 0.001
**Relapsing remitting multiple sclerosis**	0.0% (0)	0.0% (0)	0.0% (0)	0.1% (1)	0.1% (4)	0.0% (1)	0.0% (6)	0.003
**Advanced Parkinson disease**	0.0% (0)	0.0% (0)	0.0% (0)	0.3% (3)	0.5% (25)	3.1% (854)	2.6% (882)	< 0.001
**Alzheimer disease**	0.0% (0)	0.0% (0)	0.0% (0)	0.0% (0)	0.1% (4)	1.7% (465)	1.4% (469)	< 0.001
**Axial spondylarthritis**	0.0% (0)	0.0% (0)	0.4% (1)	0.3% (3)	0.1% (5)	0.1% (27)	0.1% (36)	0.268
**Rheumatoid arthritis**	0.0% (0)	0.0% (0)	1.2% (3)	1.5% (14)	2.1% (105)	2.0% (545)	2.0% (667)	0.569
**Diabetes mellitus – neonatal**	0.0% (0)	0.0% (0)	0.0% (0)	0.0% (0)	0.0% (1)	0.0% (0)	0.0% (1)	0.330
**Gestational diabetes mellitus**	0.0% (0)	0.0% (0)	0.4% (1)	0.1% (1)	0.0% (0)	0.0% (0)	0.0% (2)	< 0.001
**Diabetes mellitus – type 1**	0.0% (0)	0.0% (0)	0.4% (1)	0.1% (1)	0.1% (6)	0.1% (20)	0.1% (28)	0.478
**Diabetes mellitus – type 2**	0.0% (0)	9.7% (7)	7.5% (18)	14.8% (139)	27.3% (1360)	37.6% (10,332)	35.1% (11,856)	< 0.001
**Diabetes mellitus – insulin deficiency after medical procedures**	0.0% (0)	2.8% (2)	2.1% (5)	2.3% (22)	1.8% (88)	2.0% (557)	2.0% (674)	0.783
**Diabetes mellitus – other types**	0.0% (0)	1.4% (1)	1.2% (3)	0.2% (2)	0.8% (42)	0.8% (229)	0.8% (277)	0.388
**Thromboembolic disease – treatment in cancer patients**	0.0% (0)	0.0% (0)	0.0% (0)	0.0% (0)	0.0% (2)	0.0% (2)	0.0% (4)	0.551
**Treatment of venous thromboembolic disease**	0.0% (0)	0.0% (0)	0.0% (0)	0.4% (4)	0.2% (8)	0.3% (90)	0.3% (102)	0.374
**Thromboembolic disease – prevention in cancer patients**	0.0% (0)	0.0% (0)	0.0% (0)	0.1% (1)	0.0% (2)	0.1% (14)	0.1% (17)	0.974

[a] Quartiles: 25th, 50th, and 75th percentiles; [b] mean ± standard deviation.

Table S4. Descriptive statistics of individuals registered in the COVID-19 national digital registry, stratified by the number of vaccine doses**.**

	**Not vaccinated** *n* **= 1,952,355**	**Two doses** *n* **= 1,146,314**	**Three or more doses** *n* **= 1,788,920**	**Combined** *n* **= 4,887,589**	***p* value**
**Age (years)**	14.0 31.0 49.0[a] (33.7 ±21.6)[b]	22.0 36.0 50.0[a] (37.9 ±18.8)[b]	42.0 54.0 67.0[a] (54.9 ±16.8)[b]	25.0 43.0 58.0[a] (42.4 ±21.6)[b]	< 0.001
**Age group: 0–19**	35.3% (690 080)	20.3% (232 550)	0.1% (1069)	18.9% (923 699)	< 0.001
**20–34**	18.8% (367 290)	27.6% (316 889)	12.2% (218 985)	18.5% (903 164)	
**35–44**	14.0% (273 255)	17.4% (199 553)	17.1% (305 615)	15.9% (778 423)	
**45–54**	13.4% (261 167)	15.4% (176 266)	20.8% (372 692)	16.6% (810 125)	
**55–69**	12.0% (235 038)	12.8% (146 914)	28.1% (503 427)	18.1% (885 379)	
**70+**	6.4% (125 525)	6.5% (74 142)	21.6% (387 132)	12.0% (586 799)	
**Sex: male**	50.6% (988 145)	46.7% (535 657)	45.4% (812 575)	47.8% (2 336 377)	< 0.001
**Mortality rate due to COVID-19**	1.2% (23 606)	0.4% (4721)	0.3% (5413)	0.7% (33 740)	< 0.001
**Number of vaccination doses before last positive**	0.0 0.0 0.0[a] (0.1 ±0.3)[b]	2.0 2.0 2.0[a] (2.0 ±0.0)[b]	3.0 3.0 3.0[a] (3.1 ±0.3)[b]	0.0 2.0 3.0[a] (1.6 ±1.3)[b]	< 0.001
**Number of comorbidities: 0**	80.0% (1 562 012)	76.7% (879 756)	48.9% (875 649)	67.9% (3 317 417)	< 0.001
**1**	9.6% (187 736)	11.5% (132 242)	20.1% (359 163)	13.9% (679 141)	
**2**	5.6% (108 649)	6.4% (73 256)	15.8% (282 076)	9.5% (463 981)	
**3**	3.1% (60 636)	3.5% (39 988)	9.9% (176 342)	5.7% (276 966)	
**4**	1.2% (24 013)	1.3% (15 138)	3.9% (69 878)	2.2% (109 029)	
**5**	0.4% (7250)	0.4% (4518)	1.1% (20 115)	0.7% (31 883)	
**6**	0.1% (1718)	0.1% (1169)	0.3% (4735)	0.2% (7622)	
**7**	0.0% (341)	0.0% (247)	0.1% (962)	0.0% (1550)	
**Number of comorbidities**	0.0 0.0 0.0[a] (0.4 ±0.9)[b]	0.0 0.0 0.0[a] (0.4 ±0.9)[b]	0.0 1.0 2.0[a] (1.0 ±1.3)[b]	0.0 0.0 1.0[a] (0.6 ±1.1)[b]	< 0.001
**Year: 2020**	4.0% (78 844)	0.0% (4)	0.0% (6)	1.6% (78 854)	< 0.001
**2021**	27.4% (534 223)	18.7% (214 119)	1.3% (24 017)	15.8% (772 359)	
**2022**	67.4% (1 315 408)	79.4% (910 110)	94.3% (1 686 644)	80.0% (3 912 162)	
**2023**	1.2% (23 879)	1.9% (22 081)	4.4% (78 253)	2.5% (124 213)	
**Wave: Original/Alpha**	12.9% (251 148)	0.3% (3129)	0.0% (13)	5.2% (254 290)	< 0.001
**Delta**	15.3% (299 326)	10.9% (125 149)	0.3% (5092)	8.8% (429 567)	
**Omicron**	71.8% (1 401 880)	88.8% (1 018 036)	99.7% (1 783 815)	86.0% (4 203 731)	

**COPD**	1.4% (27 333)	1.5% (16 877)	3.7% (65 626)	2.2% (109 836)	< 0.001
**Ulcerative colitis**	0.2% (3750)	0.3% (2919)	0.4% (7683)	0.3% (14 352)	< 0.001
**Peptic ulcer**	0.2% (2936)	0.2% (1993)	0.3% (5729)	0.2% (10 658)	< 0.001
**Dyslipidaemia**	14.1% (276 109)	16.8% (192 442)	39.8% (712 673)	24.2% (1 181 224)	< 0.001
**Dyslipidaemia – PCSK9 inhibitors**	0.0% (348)	0.0% (266)	0.1% (1050)	0.0% (1664)	< 0.001
**Hypertension**	10.9% (212 448)	12.3% (140 839)	31.0% (554 323)	18.6% (907 610)	< 0.001
**Cardiac failure**	0.5% (9358)	0.5% (5696)	1.2% (21 186)	0.7% (36 240)	< 0.001
**Atrial fibrillation**	1.3% (25 338)	1.4% (15 707)	3.7% (67 022)	2.2% (108 067)	< 0.001
**Coronary artery disease**	2.4% (46 012)	2.5% (29 003)	7.0% (125 187)	4.1% (200 202)	< 0.001
**Relapsing remitting multiple sclerosis**	0.0% (281)	0.0% (221)	0.0% (637)	0.0% (1139)	< 0.001
**Advanced Parkinson disease**	0.1% (2183)	0.1% (1299)	0.3% (4577)	0.2% (8059)	< 0.001
**Alzheimer disease**	0.1% (1367)	0.1% (849)	0.2% (2728)	0.1% (4944)	< 0.001
**Axial spondylarthritis**	0.1% (2884)	0.2% (2000)	0.3% (6032)	0.2% (10 916)	< 0.001
**Rheumatoid arthritis**	0.4% (7232)	0.4% (5077)	1.1% (20 460)	0.7% (32 769)	< 0.001
**Diabetes mellitus – neonatal**	0.0% (8)	0.0% (8)	0.0% (3)	0.0% (19)	0.079
**Gestational diabetes mellitus**	0.2% (3738)	0.3% (3175)	0.3% (4832)	0.2% (11 745)	< 0.001
**Diabetes mellitus – type 1**	0.0% (132)	0.0% (83)	0.0% (287)	0.0% (502)	< 0.001
**Diabetes mellitus – type 2**	5.1% (100 010)	5.7% (65 223)	13.9% (247 824)	8.5% (413 057)	< 0.001
**Diabetes mellitus – insulin deficiency after medical procedures**	0.3% (6321)	0.4% (4108)	0.6% (11 055)	0.4% (21 484)	< 0.001
**Diabetes mellitus – other types**	0.2% (3166)	0.2% (2366)	0.4% (7082)	0.3% (12 614)	< 0.001
**Thromboembolic disease – treatment in cancer patients**	0.0% (22)	0.0% (14)	0.0% (54)	0.0% (90)	< 0.001
**Treatment of venous thromboembolic disease**	0.0% (944)	0.0% (438)	0.1% (1470)	0.1% (2852)	< 0.001
**Thromboembolic disease – prevention in cancer patients**	0.0% (69)	0.0% (37)	0.0% (171)	0.0% (277)	< 0.001

[a] Quartiles: 25th, 50th, and 75th percentiles; [b] mean ± standard deviation.
